# Target and Cell Therapy for Atherosclerosis and CVD

**DOI:** 10.3390/ijms241210308

**Published:** 2023-06-18

**Authors:** Yuliya V. Markina, Tatiana V. Kirichenko, Taisiya V. Tolstik, Anastasia I. Bogatyreva, Ulyana S. Zotova, Vadim R. Cherednichenko, Anton Yu. Postnov, Alexander M. Markin

**Affiliations:** 1 Petrovsky National Research Center of Surgery, Moscow 119991, Russia; t-gorchakova@mail.ru (T.V.K.); taya0077@mail.ru (T.V.T.); nastya.bogatyreva.96@mail.ru (A.I.B.); ulyusa000@gmail.com (U.S.Z.); vadik2222111@gmail.com (V.R.C.); anton-5@mail.ru (A.Y.P.); alexander.markin.34@gmail.com (A.M.M.); 2 Peoples’ Friendship University of Russia named after Patrice Lumumba (RUDN University), Moscow 117198, Russia

**Keywords:** cell therapy, atherosclerosis, CVD

## Abstract

Cardiovascular diseases (CVD) and, in particular, atherosclerosis, remain the main cause of death in the world today. Unfortunately, in most cases, CVD therapy begins after the onset of clinical symptoms and is aimed at eliminating them. In this regard, early pathogenetic therapy for CVD remains an urgent problem in modern science and healthcare. Cell therapy, aimed at eliminating tissue damage underlying the pathogenesis of some pathologies, including CVD, by replacing it with various cells, is of the greatest interest. Currently, cell therapy is the most actively developed and potentially the most effective treatment strategy for CVD associated with atherosclerosis. However, this type of therapy has some limitations. In this review, we have tried to summarize the main targets of cell therapy for CVD and atherosclerosis in particular based on the analysis using the PubMed and Scopus databases up to May 2023.

## 1. Introduction

Cardiovascular diseases (CVD), in particular ischemic heart disease and stroke, are the leading cause of mortality worldwide, and are responsible for about 18 million deaths per year [1]. According to estimations from the Global Burden of Disease Study 2019, CVD burden continues to rise in developed as well as in developing countries, moreover last years the rate of CVD has begun to increase in some high-income locations where it was previously declining [2]. One of the main reasons for CVDs is atherosclerosis, a chronic disease of the arterial wall characterized by the narrowing of the arteries associated with the formation of atherosclerotic plaques [3]. Mortality from atherosclerosis is associated with cardiovascular complications due to plaque rupture, which results in the formation of blood clots, leading to acute coronary syndrome or stroke [4]. CVD therapy is often started only after the onset of clinical symptoms (angina pectoris, elevated blood pressure, frequent headaches, etc.) and is mainly aimed at eliminating the symptoms, but not the cause of the disease. In this connection, the problem of timely prevention and treatment of CVD, including atherosclerosis, remains extremely relevant [5]. To date, there have been a sufficient number of developments for the pathogenetic therapy of atherosclerosis, and cell therapy is of the greatest interest among them [6]. Cell therapy is a method aimed at eliminating the mechanisms underlying the onset and progression of diseases by replacing them with various cells [7]. In the context of this therapy, stem cells (SC), genetically modified, primary, progenitor cells, and pericytes are used [8]. These cells can be grown in vitro or transplanted from patient tissues to induce self-healing, by intravenous injection or transplantation into the affected area [9]. Cell therapy can promote the regeneration of damaged cells in an organ or tissue. The most successful tool for cell therapy is stem cells, both induced pluripotent stem cells, and embryonic stem cells, which have the ability to migrate to the site of damage, differentiate and replace damaged cells, and secrete trophic factors (growth factors, morphogens, chemokines, cytokines, extracellular vesicles, and glycosaminoglycans) to repair damaged tissues. The development of cell therapy opens the opportunity to reduce the need for donor organs and immunosuppressive treatment [9].

The types of cells used as targets for cell therapy depend on the type of disease. Myosymplasts and myosatellitocytes act as target cells in the treatment of skeletal muscle diseases [10]; in the treatment of skin diseases—keratinocytes and dendritic cells, rarely melanocytes, Langerhans cells, Merkel cells and intraepidermal T-lymphocytes [11]; in cancer therapy—tumor cells (oncocytes); in CVD therapy—cardiomyocytes and myocytes [12]. Today, in cell therapy, a single dose of stem cells (SCs) is most often used, but in the treatment of cardiovascular diseases, SCs do not always survive after the first transplantation. It was shown in experiments on rodents, that 1000 of 100,000 cardiac progenitor cells remained 35 days after intramyocardial and intracoronary administration [13]. In this regard, modifications of cell therapy such as intravenous cell therapy, repeated cell therapy, the immunomodulatory effect of cell therapy, and the use of new cell types are actively developing. In addition, the development of the cultivation of organs in vitro aimed to create viable organs for humans is considered a very promising direction of cell therapy [14]. The development of this area of cell therapy is based on the ability of cells to self-organize into complex and functional structures, which leads to the spontaneous formation of a large ordered structure from independent cellular components as a result of growing the “rudiments” of organs in vitro using organoids, multicellular structures containing different types of cells and tissue layers presented in an adult organ, and embryoids, three-dimensional aggregates of cells containing all three germ layers necessary for the development of organs and tissues [15]. For example, a cluster of dissociated mouse embryonic stem cells (ESCs) cultured in vitro has been shown to spontaneously form the optic cup, exposing all layers of the retina, when using a medium containing low levels of growth factors and enriched with basement membrane proteins [16]. This structure, called an organoid, shows a strong similarity to in vivo tissue. In addition, cardiovascular organelles capable of contraction, as well as liver cells, kidney organelles, retinal organelles, and many others, have been obtained. Their creation is associated with modifications of 3D culturing conditions and imitation of supposed in vivo signaling events with external factors [17]. It should be noted that the creation of organelles is extremely important for modern science and medicine, but further research in this area and the improvement of these methods are necessary.

To date, stem cell therapy is a promising method for the treatment of various pathologies, including diseases of the cardiovascular system. In this review, we tried to summarize the available data on cell therapy for atherosclerosis and a number of other cardiovascular pathologies.

## 2. Target Therapy for Atherosclerosis

### 2.1. The Pathogenesis of Atherosclerosis at the Cellular Level

Atherosclerosis is a chronic inflammatory disease that affects the arterial wall and is based on the processes of lipid accumulation and immune responses. The pathogenesis of this disease is quite complex, from the early development of an atherosclerotic lesion to plaque formation [18]. At the initial stage of atherogenesis, there is a massive accumulation of multiple-modified atherogenic low-density lipoproteins (LDL), including oxidized LDL, in the subendothelial space of the intima of the arterial wall, which triggers innate and adaptive immune responses and leads to the activation of monocytes, endothelial cells (ECs), smooth muscle cells (SMCs) and platelets [19,20,21]. As a result, circulating monocytes absorb modified LDL and further differentiate into macrophages in the vascular intima and transform into foam cells [22,23]. Classical monocytes with the CD14^+^CD16^−^ phenotype is the predominant subpopulation in the blood and are recruited to inflammatory foci, including atherosclerotic plaques, while non-classical monocytes with the CD14+CD16+ phenotype usually remain in the bloodstream to control the integrity of the endothelium [24]. Foam cells formed from macrophages produce pro-inflammatory cytokines that support the formation of persistent inflammation that stimulates macrophage apoptosis. A necrotic nucleus is formed due to the accumulation of apoptotic cells in the atherosclerotic plaque, which leads to the instability of plaque and the possibility of its rupture [25]. It should be noted that an important feature of macrophages is their plasticity, which consists of their ability to adapt the immune response to changes in the microenvironment, thereby regulating inflammation [26]. Depending on the pathways of activation and physiological functions, macrophages are classified into pro-inflammatory (M1-phenotype), anti-inflammatory (M2-phenotype), and unpolarized M0-phenotype [27]. M0 macrophages can polarize into M1 macrophages under stimulation with lipopolysaccharide (LPS) and interferon-γ (IFN-γ), as well as into the M2 phenotype under the action of cytokines IL-4 and IL-13 [26]. Differences in the functions of M1 and M2 macrophages lie in the production of various cytokines. Thus, M1 macrophages secrete pro-inflammatory factors IL-1β, IL-6, and TNF-α, while M2 macrophages secrete anti-inflammatory factors such as IL-1 receptor agonist, IL-10, and collagen [28]. Therefore, M1 macrophages are involved in the development of inflammation and contribute to the destruction of tissues, while M2 macrophages possess anti-inflammatory properties and contribute to the resolution of inflammation. Since macrophages play a key role in the pathogenesis of atherosclerosis, it can be assumed that targeting these cells may be a promising therapeutic approach for atherosclerosis therapy.

Some studies on the pathogenesis of atherosclerosis at the cellular level revealed that there is a violation of the structure and function of ECs lining the inner surface of blood vessels in atherosclerotic patients [29]. ECs perform many functions, including the regulation of vascular tone, the production of angiogenic factors, and the maintenance of the anticoagulants balance and blood coagulation [30]. In the presence of risk factors such as high blood levels of cholesterol, smoking, and diabetes, ECs are exposed to damaging effects that lead to impaired function. One of the main functions of ECs is the synthesis of nitric oxide (NO), which regulates vascular tone, reduces the adhesion of platelets and monocytes, and can prevent the development of atherosclerosis [31]. NO synthesis can be reduced in damaged ECs, which stimulates the development of atherosclerosis. On the other hand, dysfunctional ECs can lead to increased activation of endothelial NO synthase (eNOS) and increased production of NO, which promotes vasodilation and increased blood flow [32]. However, experimental and clinical studies have shown that overproduction of NO can lead to the development of vascular dysfunction, as well as other pathological processes such as atherosclerosis, hypertension, etc. [33]. Thus, the balance between mechanical and biochemical regulators is critical for maintaining normal endothelial function.

Another type of cell that plays an important role in the development of atherosclerotic lesions is SMCs, which make up the main structure of the vessel wall, and provide its tone and elasticity. The structure and functions of SMCs change under conditions of chronic hypertension, endothelial damage or hypercholesterolemia. One of the key stages in the development of atherosclerosis is the formation of an atherosclerotic plaque. During this process, SMCs proliferate and migrate to the intima, where produce an extracellular matrix containing structural proteins, including collagen and elastin, which leads to vessel wall thickening [34]. SMCs may also be involved in inflammatory processes, vascular hypertrophy, and remodeling. SMCs can produce cytokines and growth factors, which lead to the recruitment of inflammatory cells to the sites of lipid accumulation in the vessel wall and the formation of atherosclerotic plaque [35]. These pathogenetic mechanisms of atherogenesis associated with the participation of SMCs represent potential targets for the treatment of atherosclerosis.

It is believed that inflammation is not only an important predictor of atherosclerosis development but also an important factor in cell aging [36]. Cellular aging is an irreversible stoppage of the cell cycle, accompanied by the production of pro-inflammatory cytokines by monocytes/macrophages, such as interleukin (IL)-1α, IL-1β, IL-6, IL-8, IL-18, CCL-2, TNF-α, metalloproteinases (MMPs), and other factors [37]. In addition, senescent cells are considered biomarkers of cardiovascular risk [38]. Cellular senescence is observed in the initial stages of atherosclerosis and affects different types of cells: ECs, SMCs, monocytes, fibroblasts, and T-cells [39]. In this regard, the suppression of cellular aging processes is of great interest as a new method of amelioration of atherosclerosis progression, aimed at inflammation reduction and stabilization of atherosclerotic plaques.

**Figure 1:**
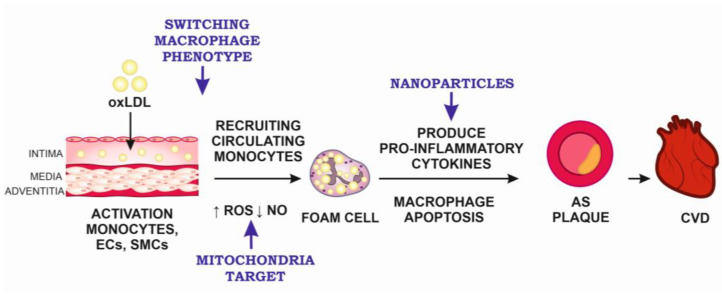
The main targets for anti-atherosclerotic therapy. AS, atherosclerosis; CVD, cardiovascular disease; ECs, endothelial cells; NO, nitric oxide; oxLDL, oxidized low-density lipoproteins; ROS, reactive oxygen species; SMCs, smooth muscle cells.

The possible targets for anti-atherosclerotic therapy are presented in Figure 1 with respect to the most important pathogenetic mechanisms of atherosclerosis development from the initial stage of atherogenesis such as accumulation of the modified LDL in the arterial wall and activation of immune cells to the formation of severe atherosclerotic plaque.

### 2.2. Monocyte Activation As a Target for Atherosclerosis Therapy

Monocytes/macrophages play a key role in the inflammatory process in atherosclerosis, promoting the secretion of inflammatory mediators and altering lipid metabolism [40]. At present, there is no doubt that the polarization of macrophages plays a fundamental role in atherogenesis. The imbalance of the M1/M2 ratio affects the development of atherosclerosis, since pro-inflammatory M1 macrophages promote atherosclerosis progression while anti-inflammatory M2 macrophages stimulate tissue repair. In this regard, regulation of macrophage polarization is an extremely promising therapeutic strategy for the prevention of atherosclerotic lesions development [41]. So, the most widely used anti-atherosclerotic preparations from the group of HMG-CoA reductase inhibitors were demonstrated to switch the macrophages phenotype promoting anti-inflammatory condition. For example, the most popular in clinical practice preparation Rosuvastatin affects the M2 macrophage polarization by reduction of iNOS expression in the arterial wall of ApoE-/- mice and stimulation of the M2 markers expression such as arginine-1, CD206 as well as ABCA1 and ABCG1 genes [42]. A number of anti-diabetic preparations, such as an inhibitor of dipeptidyl peptidase 4 (DPP-4) Sitagliptin and sodium-glucose co-transporter 2 (SGLT2) inhibitor Dapagliflozin, have been shown to stimulate the anti-inflammatory polarization of macrophages, and thus prevent the formation of atherosclerotic lesions [43,44].

In addition to the described preparations, the anti-atherosclerotic efficacy of various biomolecules that possess an anti-inflammatory effect by switching the macrophage phenotype is being actively studied in animal models. In particular, an endogenous fatty acid mediator Palmitoylethanolamide was shown to decrease the markers of M1 macrophages phenotype in ApoE-/- mice and at the same time reduced the size of atherosclerotic plaques and stimulated plaque stability by reduction of macrophage accumulation and necrotic core size and stimulation of collagen deposition [45]. In the other study, it was demonstrated that the plasma protein Kallistatin significantly inhibited the formation of atherosclerotic lesions and reduced inflammation in atherosclerotic plaques in ApoE-/- mice since it upregulated the expression of M2 macrophage marker IL-10 and downregulated the expression of M1 macrophages marker MCP-1 and inducible NO synthase [46].

In the mitochondria of monocytes/macrophages, either glycolysis or oxidative phosphorylation (OXPHOS) can occur depending on the metabolic inhibitors. Glycolysis occurs in M1 macrophages, while M2 macrophages are characterized by fatty acid oxidation processes in mitochondria [47]. In most cases, the main factor in the activation of monocytes/macrophages is LPS, the main component of the cell walls of Gram-negative bacteria. In response to LPS-induced stimulation, monocytes/macrophages produce key inflammatory mediators, inflammatory cytokines, and chemokines, triggering inflammation and promoting atherosclerosis progression [48]. LPS has been shown to increase glucose uptake and glycolysis in monocytes. Signal transduction requires the participation of extracellular proteins such as CD14 (glycosylphosphatidylinositol (GPI)-anchored protein) and LPS-binding protein (LBP) [49]. CD14 binds to heteromeric G proteins, thereby protecting cells from LPS exposure [50]. Studies on animal models demonstrated the protective effect of G protein-binding peptide against LPS-induced mortality, hence there is a functional link between CD14 and associated intracellular signaling molecules [51]. Another study showed that ApoE-/- mice exposed to ultra-low doses of LPS had higher levels of circulating pro-inflammatory monocytes compared to control mice [52]. The increased level of the inflammatory chemotaxis receptor CCR5, one of the main monocyte markers associated with atherosclerosis was observed in ApoE-/- mice [53]. Also, a decrease in SR-B1 (scavenger receptor, class B type 1) level, a modulator of inflammation and metabolism in monocytes was demonstrated in ApoE-/- mice. In another study, a decrease in the expression level of SR-B1 was observed in monocytes isolated from the aorta of mice treated with ultra-low doses of LPS compared to control animals [54].

### 2.3. Mitochondrial Therapy for Atherosclerosis

Violation of energy metabolism is an important factor in the development of atherosclerosis. The main processes of cellular energy metabolism occur in mitochondria [55]. Mitochondria are involved in the production of reactive oxygen species (ROS), which are involved in cell death, calcium regulation, and the formation of foam cells in atherosclerosis [56]. Mitochondrial dysfunction can lead to cellular senescence, inflammation, and apoptosis, which accompany the development of atherosclerosis [57]. Mitochondrial mutations are important factors leading to dysfunction of oxidative phosphorylation and energy metabolism, as well as endothelium damage [58]. Thus, mitochondria can be one of the main targets for the development of new preparations for the treatment of atherosclerosis and cardiovascular disease [59].

To date, there are a number of anti-atherosclerotic therapeutic agents targeting mitochondria. The most commonly used in CVD treatment preparations, such as aspirin, statins, and renin-angiotensin system inhibitors, possess pleiotropic antioxidative effects while also targeting mitochondrial ROS, thus improving mitochondrial functions and supporting beneficial anti-atherosclerotic activity [60]. Various preparations based on natural products with antioxidant action may also be used to improve mitochondrial functions in complex anti-atherosclerotic treatments [61]. It was shown that the natural preparation of luteolin contributed to the elimination of symptoms of oxidative stress in atherosclerosis due to its antioxidant effect [62]. Several studies have demonstrated that resveratrol promotes mitochondrial fusion and improves metabolism in endothelial cells by maintaining mitochondrial membrane proteins and reducing ROS production [63]. The preparation of ilexgenin A possessed an inhibitory effect on the expression of dynamin 1-related protein (DRP1), which is a regulator of mitochondrial fission, that can reduce the production of ROS and inflammatory mediators, and also improve endothelial dysfunction, thereby preventing atherogenesis [64].

### 2.4. Other Targets for Atherosclerosis Therapy

Nanomedicine aimed at the selective delivery of small molecules using nanoparticles (NP) to macrophages of atherosclerotic plaque is an actively developing strategy for the treatment of atherosclerosis. Targeted therapy is carried out by attaching peptides and other ligands to the surface of functional NP antibodies [65]. A large number of nanoparticle platforms have been developed for the delivery of RNA interference therapeutics, including small interfering RNAs (siRNAs). Clinical trials of NP-based siRNA delivery have demonstrated that preparations based on soy were effective for plaque stabilization by downregulating genes encoding monocyte-associated proteins and factors, including CD14, CD68, LYZ, CCL2, CCL3, IL1B, which destabilize plaque in damaged macrophages [66]. In addition, NPs can be used in diagnostics for accurate visualization of plaques in vessels [67]. In experiments on the C57BL/6 mouse model, intravenous administration of nanoparticle-encapsulated IL-10 reduced the production of the pro-inflammatory cytokine IL-1β in atherosclerotic lesions, resulting in a reduction of plaque size. These data suggest that the delivery of nanoparticle-based cytokines to reduce inflammation may be an effective therapeutic strategy for the treatment of atherosclerosis.

The use of natural and synthetic lipoproteins can also be an effective tool in nanomedicine for targeted therapy of atherosclerosis. Lipoproteins are endogenous particles that transport fats and cholesterol in the human body. High-density lipoproteins are the most important type of lipoproteins that possesses a protective mechanism based on the removal of excess cholesterol from peripheral tissues, especially from lipid-rich macrophages during atherogenesis. In this regard, artificial HDL nanoparticle-based therapy aimed to increase HDL levels can help inhibit lipid deposition and reduce the development of atherosclerotic lesions in human arteries [68]. However, a nanoparticle-based therapy to increase HDL levels should be further studied to assess its effectiveness in terms of atherosclerosis progression. In particular, inhibitors of major modulators of blood HDL—cholesteryl ester transfer protein (CETP) did not proven their efficacy in clinical trials of CVD at present, but this therapeutic strategy is still being studied [69].

## 3. Cell Therapy for Atherosclerotic CVDs

### 3.1. Cell Therapy for Myocardial Infarction

**Figure 2:**
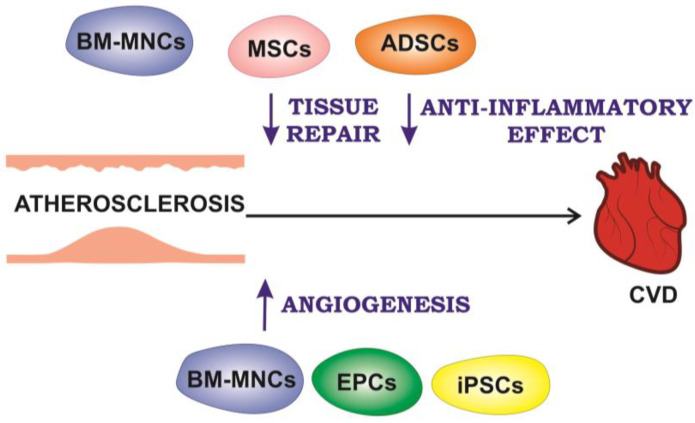
Main approaches to CVD cell therapy; BM-MNCs—bone marrow mononuclear cells; ADSCs—adipose-derived stem cells; EPCs—endothelial progenitor cells; iPSCs—pluripotent stem cells; MSCs—mesenchymal stem cells.

Myocardial infarction (MI) is a CVD, representing irreversible myocardial injury leading to heart failure and sudden death [70]. Despite the advances in revascularization of patients with acute coronary syndrome, myocardial infarction still leads to heart remodeling resulting in a significant increase in heart failure progression, and cell therapy is being considered as a potential approach to regenerate and preserve myocardial viability. Experimental cell therapy approaches are based on various cell types, including bone marrow mononuclear cells (BM-MNCs), mesenchymal stem cells (MSCs), resident cardiac or progenitor stem cells, endothelial progenitor cells (EPCs), stem cells from other tissues such as adipose tissue, embryonic stem cells (ESCs), induced pluripotent stem cells (iPSCs) and myoblasts, with and without genetic modification to increase functionality [71]. Figure 2 presents different types of cells used for cell therapy of myocardial infarction, peripheral arterial disease (PAD), and their beneficial effects on the development of atherosclerosis-associated CVD, which are discussed below.

The identification of a reliable population of cardiac stem cells (CSCs) is an important step in the development of cell therapy for patients with heart failure. Among the various markers used to identify resident CSCs, c-kit plays a significant role [72]. Cardiac c-kit-positive (c-kit+) cells were the first population of putative CSCs described as negative for the bloodline marker (Lin-) in the adult rat heart, along with having self-renewing, clonogenic, and multipotent characteristics [73,74]. C-kit+ cardiac cells also proved to be necessary and sufficient for myocardial regeneration after cardiac injury in rats and mice [75]. It has been revealed that c-kit-expressing cardiac cells are in fact a subset of ECs in the developing and adult mouse hearts. C-kit+ cells rarely express the cardiomyocyte marker Nkx2.5 or the differentiated cardiomyocyte marker cardiotroponin T (cTnT). C-kit+ cells don’t become cardiomyocytes during tissue damage or after injury. These results suggest that c-kit+ cells in the mammalian heart are actually endothelial cells and not CSCs [72].

Recent studies have shown that there are separate subpopulations of macrophages in the heart, which in different ways contribute to the development and recovery of the myocardium after myocardial infarction [76]. Neutrophils, pro-inflammatory monocytes, and M1 macrophages infiltrate the myocardium determining the inflammatory response immediately after myocardial infarction. The main goal of this response is to remove necrotic cells from the area of myocardial infarction [77]. So, in the first 3–4 days after myocardial infarction, most macrophages in the heart exhibit the pro-inflammatory M1 phenotype, followed by a rapid increase of M2 macrophages in the 5–7th days [78]. M2 macrophages are able to secrete anti-inflammatory cytokines (for example, IL-1ra, IL-10, TGF-β families), which contribute to the resolution of inflammation as well as enhance the formation of connective tissue, activating fibroblasts due to the secretion of profibrotic cytokines and affecting the balance between matrix metalloproteinase (MMP) and tissue inhibitor of metalloproteinase (TIMP). In addition, M2 macrophages are involved in various stages of neovascular formation. These data strongly suggest that a further increase in M2 macrophages leads to increased myocardial recovery and an improved prognosis after infarction [76].

Cell transplantation has attracted attention as a new treatment strategy for myocardial infarction [79]. In this regard, BM-MNCs became an attractive research target as a source of donor cells due to the ease of obtaining a large number of autologous cells, which include several types of progenitor stem cells. BM-MNCs have the ability to repair the heart after myocardial infarction [80]; however, the therapeutic efficacy of this approach in clinical trials is variable [76].

BM-MNCs are a natural source of M2-like reparative macrophages [76]. Unlike BM-MNCs foreign to cardiac tissue, reparative macrophages represent cells that naturally settle in an injured heart, demonstrating improved survival in the heart. The innate ability of macrophages to migrate and settle in damaged tissue may also contribute to the efficient functional engraftment of transplanted cells in the damaged heart compared to BM-MNCs [81]. It was also demonstrated that BM-MNC therapy reduces inflammation-associated parameters [82].

MSC-based therapies are currently being used to treat both acute myocardial infarction and chronic ischemic cardiomyopathy. MSCs are thought to work by activating endogenous tissue repair via paracrine signaling and also exhibit immunomodulatory properties, reducing immune-mediated damage after infarction [83]. Additionally, MSCs possess an antifibrotic effect and cause reverse left ventricular remodeling in preclinical models [84]. In this regard, a strategy called in vivo priming has been developed. BM-MSCs are primed in vivo in a heart with myocardial infarction by genetically engineered MSCs expressing hepatocyte growth factor (HGF-eMSC) that are encapsulated within an epicardially implanted cardiac 3D patch. HGF-eMSC primed BM-MSCs demonstrate improved vasculogenic potential and cell viability, which ultimately enhance vascular regeneration and improve cardiac function after myocardial infarction [85].

The discovery of extracellular vesicles, including exosomes, as a key component of the beneficial function of stem cells has raised hopes that they can be used to advance cell regenerative therapies aimed at repairing the heart. Exosomes secreted by stem cells are membranous bionanovesicles loaded with various proteins, lipids, and nucleic acids. They have been found to possess antiapoptotic, antifibrotic as well as proangiogenic effects, which are critical for the regeneration of damaged myocardial function [86]. ESC-derived mouse exosomes have been shown to promote endogenous recovery and preservation of cardiac function upon intramyocardial administration immediately after ligation of the left anterior descending artery in a mouse model of infarction [87]. The beneficial effects observed with ESC-derived exosomes are mediated, at least in part, by the transfer of exosome miR-294 [86].

Given all the limitations and disadvantages, the idea of realizing efficient regeneration of cardiac tissue using only one type of cell is likely to be challenging. Depending on further mechanistic and clinical understanding, multi-stage approaches that include various stem and stromal cells, paracrine factors, and specifically bioengineering tools for future cardio-regenerative medicine will prove to be the most effective [88].

### 3.2. Cell Therapy for Peripheral Arterial Disease

PAD is a clinical manifestation of atherosclerotic lesions of the peripheral arteries of the lower extremities with subsequent stenosis and occlusion [89]. The most severe form of PAD manifestation is critical limb ischemia (CLI), which leads to limb loss and increases CVD mortality [90]. PAD affects more than 200 million patients worldwide [91]. Traditional cardiovascular risk factors, including older age, smoking, diabetes mellitus, hypertension, and hyperlipidemia, are strongly associated with an increased risk of the disease [92]. This disorder rarely occurs before the age of 50 years, but affects up to 20% of people aged 80 years and older [93]. The classic symptoms of PAD are intermittent claudication, atypical pain in the legs associated with walking and ameliorated at rest, and limb ischemia; in addition, the disease may be asymptomatic [94]. Clinical manifestations of PAD are associated with blood flow restriction, resulting in an imbalance between blood supply and blood demand [95]. Repetitive limb ischemia triggers ROS generation caused by mitochondrial dysfunction in skeletal myocytes. ROS induces apoptosis of skeletal myocytes, which leads to a decrease in the mass of skeletal muscles, the appearance of fatty infiltration, dysfunction of peripheral nerves, and fibrosis [96]. Given the severe symptoms and consequences of PAD, as well as the limited treatment options for the severe disease, new therapeutic strategies are needed to prevent the development and progression of the disease, and to treat life-threatening complications. Experimental studies have shown that cell therapy can be a promising approach for patients with PAD [6].

The main aim of PAD treatment is to restore blood circulation in the affected limb area. Theoretically, an increase in blood flow can be achieved by improving the regeneration and function of the vessels supplying blood to the ischemic tissue [97]. Angiogenesis is defined as the growth and proliferation of blood vessels from existing vascular structures [98]. The main process of angiogenesis is the germination of ECs from pre-existing capillaries under the regulation of angiogenic factors such as vascular endothelial growth factor (VEGF) and fibroblast growth factor (FGF) generated by ischemic tissue. PAD is characterized by a pathological form of angiogenesis, which consists of the germination of capillaries. In this case, the angiogenic response to ischemia is insufficient to meet tissue needs [99]. Under hypoxia conditions, the transcription factor hypoxia-inducible factor 1 (HIF-1) is activated, since its subunit HIF-1α has a degradation domain depending on oxygen tension [100]. When oxygen levels are low, HIF-1α stabilizes, moves to the nucleus, and activates target genes including VEGF, FGF, hepatocyte growth factor (HGF), platelet growth factor (PDGF), developmental endothelial locus-1 (Del-1), angiopoietins, and matrix metalloproteinases to facilitate angiogenesis [101]. Arteriogenesis can be considered the completion of angiogenesis development. In contrast to angiogenesis, arteriogenesis is a positive remodeling process of pre-existing collateral channels that can significantly increase blood flow [102]. In normal states, collateral canals are narrow-lumen, high-resistance vessels that provide little blood flow to their distal tissue bed [99]. When the primary artery is occluded, a pressure decrease subsequently causes a diversion of blood flow through the collaterals. The altered blood flow creates a hemodynamic stimulus, which entails an increase in the diameter and thickness of the walls of the collateral canals with the proliferation of vascular cells [103]. These processes activate the expression of chemokines and adhesion molecules by the endothelium [104]. Chemokines trigger the recruitment and attachment of circulating monocytes to activated endothelium [105]. Monocytes then pass through the endothelium to the subintimal space, where they transform into macrophages and produce inflammatory cytokines and growth factors such as transforming growth factor-β (TGFβ), tumor necrosis factor-α (TNFα), epidermal growth factor (EGF) and FGF, which promote positive vascular remodeling [102]. Patients with PAD have the possibility of arteriogenesis, but the ability to restore limb perfusion to normal levels is severely limited [99].

EPCs have been demonstrated to promote the development of new blood vessels in angiogenesis [106]. Circulating cells with angiogenic potential exit the bone marrow into the bloodstream under the influence of granulocyte-colony-stimulating factor (G-CSF) and granulocyte-macrophage-colony-stimulating factor (GM-CSF). These CD34+ circulating angiogenic cells can also express CD133, VEGFR2, and CXC chemokine receptor type 4 (CXCR-4) [107]. Angiogenic cells migrate to the ischemic tissues under the action of stromal cell-derived factor 1 (SDF-1), which binds to CXCR-4 [108]. Bone marrow-derived angiogenic cells of hematopoietic origin improve perfusion through the secretion of cytokines and metalloproteinases [109].

The first series of trials using BM-MNCs was conducted in 1999. The hypothesis was that the bone marrow is not only a reservoir of hematopoietic cells, but also contains EPCs [110]. It was assumed that EPCs promote the formation of vessels leading to angiogenesis [111]. But the double-blind, placebo-controlled, randomized JUVENTAS trial showed no significant therapeutic effects in PAD patients [112]. Further studies related to progenitor cell therapy have shown that cells isolated from the bone marrow integrate unstably into the formed vessels and play an additional role in neovascularization [113]. Endothelial colony-forming cells (ECFCs) demonstrated angiogenic properties [114]. However, the use of ECFCs is limited by low circulating levels and the loss of stem cell phenotype associated with the change to an aging phenotype in culture [115]. Taken together, these observations accelerated the development of cell therapy [116].

Stem cell therapy has become one of the main strategies for the treatment of PAD. Stem cells are capable of self-renewing and producing differentiated cells. Based on the degree of differentiation, they can be divided into totipotent, pluripotent, multipotent, oligopotent, or unipotent [117]. Bone marrow stem cells include various types of progenitor cells: multipotent adult progenitor cells, MSCs, and hematopoietic stem cells [118]. MSCs are the most actively studied type of cells among various potential candidate cells that can be used to treat patients with PAD [97]. MSCs have been found to promote angiogenesis, arteriogenesis, and terminal differentiation into vascular cells and myocytes through their paracrine-related effects [119]. It was also shown that these cells secrete antiapoptotic, antifibrotic, immunomodulatory, and chemoattractant factors, and are characterized by the ability to survive in an ischemic environment due to metabolic flexibility and resistance to ischemic stress [120]. However, the mechanism of action of the MSC remains controversial. They are believed to release cytokines that stimulate the migration of endogenous ECs. Further differentiation and proliferation of ECs in situ lead to an increase in the stocks of resident ECs [121].

Adipose-derived stem cells (ADSCs) represent a source of stem cells for therapeutic use in PAD [121]. Autologous adipose tissue contains mature adipocytes, which make up the bulk of adipose tissue, and a stromal vascular fraction (SVF), consisting of multipotent MSCs that are able to differentiate into various clones: myocytes, adipocytes, fibroblasts, osteoblasts, and chondroblasts and to regenerate damaged tissue [122,123]. In an animal model of hind limb ischemia, transplantation of ADSCs was shown to improve angiogenesis [124]. Subsequently, it turned out that ADSCs do not have the ability to differentiate into ECs or EPCs, but have similar markers as pericytes and mesenchymal cells [125]. ADSC transplantation affects therapeutic angiogenesis through the secretion of pro-angiogenic chemokines and cytokines such as SDF-1, VEGF and HGF [126].

Induced pluripotent stem cells (iPSCs) may become an alternative to human embryonic stem cells (hESCs), since there is an ethical problem associated with the use of embryos for ESCs treatment [121]. iPSCs can be reprogrammed in vitro by introducing transcription factors into the nucleus of fibroblasts, which leads to the expression of genetic markers characteristic of ESCs by somatic cells [127,128]. Fetal liver kinase-1 (Flk-1) cells derived from iPSCs were studied in a mouse model. It turned out that Flk-1 stimulates angiogenesis in the ischemic tissue of the limb due to a temporary increase in the concentration of VEGF and the direct incorporation of ECs into the ischemic tissue [129]. With further research, iPSCs may become the most promising strategy for PAD cell therapy [121].

## 4. Conclusions

Currently, cell therapy is the most actively developing and potentially the most effective therapy for atherosclerosis-associated CVD. However, it has some limitations nowadays. This is primarily due to safety issues, as cell therapy can cause serious side effects such as immune reactions and rejection, infections, thrombosis, and carcinogenesis which depend on various factors including patients’ health and the type of cells used for the CVD therapy. In addition, cell therapy is a complex process requiring precise control of many variables, and there is currently no standardized protocol for obtaining cells. This type of therapy is also expensive due to the high cost of production and the need for specialized equipment. At the same time, despite the fact that cell therapy has shown promising results in some clinical trials, it may not be effective for all patients or conditions. Thus, cell therapy for CVD treatment has been widely studied in various research studies in the last few years, however, there has been no breakthrough due to the described limitations. Nevertheless, the concept of cell therapy for CVD remains highly demanded, which is explained by the high prevalence and medical and social significance of CVD in modern society and the need to develop new therapeutic approaches for CVD. The development of new strategies for target therapy of subclinical atherosclerosis is of equal importance, since timely prevention can significantly reduce the progression of atherosclerosis and the risk of developing CVD.

## Data Availability

Not applicable.
